# The R.B.N.A. at Bay

**Published:** 1891-06-06

**Authors:** 


### Passing Topics.

### THE R.B.N.A. AT BAY.

When a man is fighting—even in a bad cause—against overwhelming odds, we do not judge him harshly if in his desperation he shows himself insensible to the demands of strict courtesy and fairness; or if, in a last wild effort, he deals his opponent a foul blow. Similarly, we should desire to complain as little as possible of the mere tone of the remarkable manifesto which certain representatives of the Royal British Nurses' Association have lately given to the world in the columns of the *Times.* It is only natural that there must be some bitterness of feeling in a body which not so long ago was organised under promising and somewhat imposing circumstances, and has so soon become discredited, and its opponents may not unfairly claim that the acrimonious personalities of surely as rude a letter as was ever publicly penned, give the measure of its supporters' despair, and, perhaps, afford evidence of impending dissolution.

We will not deny the Association method in its madness. That the Treasurer of St. Thomas's Hospital should be singled out for especial vituperation is eminently characteristic of the pretensions of the Association, and strictly consistent with its earliest attitude. The gentleman in question is a "layman," and, consequently, he is among the unspeakables. He is not even to be damned with the faint praise which would allow him the possession of good intentions. He is to be ruthlessly condemned and held up to ridicule because he dares to hold an opinion on the subject of the registration of nurses. Not only so, he is the successor of gentlemen who, we are politely told, have been commonly uncertain as to the use of aspirates, and to the logical minds of the defenders of the R.B.N.A, this is a convincing proof of incapacity. Well, possibly the Treasurer of St. Thomas's will survive, and, conceivably, he may enjoy, the denunciation even of so eminently argumentative and select a body as the R.B.N.A.; but, for our own part, we can only entertain a deep feeling of regret that the names of two gentlemen standing high in professional and general esteem should have been put to a document they surely cannot have composed, and which is not less damaging to the signatories than to the cause it is so poorly calculated to serve.

Mr. Wainwright, like the "small number of men, mostly carrying on business in the City," can afford to smile at the sneer intended to cast a doubt upon their claim to common sense and to relegate them to the position of book-keepers, and nothing more, in the hospital whose affairs they administer. Perhaps some day those who are now putting forward this demand for undiluted professionalism will awake to the fact that laymen have their rights in respect of hospital management as well as their duties, and that to affect to despise the co-operation of those whose assistance is indispensable is nothing short of fatuous. Society at large is chiefly composed of that element which the spokesmen and spokeswoman of the R.B.N.A. reckon of no account. It is laymen who will deliver the ultimate verdict by which the Association must stand or fall, and a few more effusions such as to that to which the *Times* mercifully gave the mitigated prominence of a supplemental position would certainly ensure that the verdict should be adverse.

Agricola.

